# Perioperative Dexmedetomidine attenuates brain ischemia reperfusion injury possibly via up-regulation of astrocyte Connexin 43

**DOI:** 10.1186/s12871-020-01211-7

**Published:** 2020-12-07

**Authors:** Xiaoyang Zheng, Xiaoying Cai, Fang Ye, Ying Li, Qin Wang, Zhiyi Zuo, Wenqi Huang, Zhongxing Wang

**Affiliations:** 1 Department of Anesthesiology, The First Affiliated Hospital, Sun Yat-sen University, No. 58 Zhongshan 2nd Road, Guangzhou, 510080 Guangdong People’s Republic of China; 2 Department of Pharmacology, Zhongshan School of Medicine, Sun Yat-Sen University, No. 74 Zhongshan 2nd Road, Guangzhou, 510080 Guangdong People’s Republic of China; 3 Department of Anesthesiology, University of Virginia, 1 Hospital Drive, PO Box 800710, Charlottesville, Virginia 22908-0710 USA

**Keywords:** Brain ischemia, Reperfusion injury, Dexmedetomidine, Astrocytes, Connexin 43

## Abstract

**Background:**

Astrocyte Connexin 43 (Cx43) is essential for the trophic and protective support of neurons during brain ischemia reperfusion (I/R) injury. It is believed that dexmedetomidine participates in Cx43-mediated effects. However, its mechanisms remained unclear. This study aims to address the relationship and regulation among them.

**Methods:**

Adult male Sprague-Dawley rats were allocated to the 90-min right middle cerebral arterial occlusion with or without dexmedetomidine pretreatment (5 μg/kg). Neurological functions were evaluated and brain lesions, as well as inflammatory factors (IL-1β, IL-6, TNF-α), were assessed. Ischemic penumbral cortex was harvested to determine the expression of astrocyte Cx43. Primary astrocytes were cultured to evaluate the effect of dexmedetomidine on Cx43 after oxygen-glucose deprivation.

**Results:**

Dexmedetomidine pretreatment attenuated neurological injury, brain lesions and expression of inflammatory factors (IL-1β, IL-6, TNF-α) after brain ischemia (*P* < 0.05). Astrocyte Cx43 was down-regulated by brain I/R injury, both in vivo and in vitro, which were reversed by dexmedetomidine (*P* < 0.05). This effect was mediated by the phosphorylation of Akt and GSK-3β. Further studies with LY294002 (PI3K inhibitor) or SB216763 (GSK-3β inhibitor) confirmed the effect of dexmedetomidine on astrocyte Cx43.

**Conclusions:**

Perioperative dexmedetomidine administration attenuates neurological injury after brain I/R injury, possibly through up-regulation of astrocyte Cx43. Activation of PI3K-Akt-GSK-3β pathway might contribute to this protective effect.

**Supplementary Information:**

The online version contains supplementary material available at 10.1186/s12871-020-01211-7.

## Background

Ischemic injury of the brain frequently occurs in stroke or cardiac arrest, and certain operative procedures that ultimately progress to perioperative stroke, including neurosurgery and bypass surgery [1, 2]. Both permanent and transient brain ischemia are characterized by cell death. In our study, we focused on the latter for it may exacerbate further injury by reperfusion. The high morbidity and mortality make it essential to discover its potential mechanism.

Gap junctions (GJs) are potent targets for the reperfusion injury. As key channels for the exchange of chemical and energy substrates, astrocytes GJs play a pivotal role in the trophic and protective support for neurons [3]. During brain ischemia/reperfusion (I/R), gap junctional coupling is down-regulated and accompanied by the partial accumulation of excessive reactive oxygen species (ROS) [4]. It has been demonstrated that induction of functional GJs in astrocytes could alleviate neuron damage during brain I/R [5]. Thus, activation of astrocyte GJs might be an effective way to relieve brain I/R injury.

Dexmedetomidine, an agonist of α_2_-adrenergic receptors, is attracting rising attentions for its perioperative protective effects [6]. It could relieve neuronal necrosis and inflammation during I/R, as well as regulate GJs in astrocytes [7]. Dexmedetomidine may inhibit brain ischemia/reperfusion-induced neuroinflammation via activation of AMPK or reducing excessive mitophagy and autophagy [8, 9]. It is reported that dexmedetomidine pretreatment can attenuate intestinal injury in intestinal ischemia-reperfusion rat model [10]. Also, dexmedetomidine could affect the expression of GJs in rat kidney proximal tubular cells during I/R [11]. There are a few studies suggesting that dexmedetomidine regulates GJs activity in both astrocytes and neurons [7]. However, the regulation of GJs by dexmedetomidine pretreatment in brain I/R injury remains unknown.

Connexin 43 (Cx43), the most prominently expressed GJs in astrocytes, is modulated by Akt activity. It is proven that the activation of phosphatidylinositol-4,5-bisphosphate 3-kinase (PI3K)/Akt pathway could protect against brain I/R injury [12]. Upregulation of Cx43 may be related to the protective effect of imatinib in global ischemia-reperfusion-induced cerebral injury [13]. Other researches showed dexmedetomidine remarkably reduced oxidant function and apoptosis through activating glycogen synthase kinase-3β (GSK-3β) [14]. Therefore, the purpose of this study is to investigate whether dexmedetomidine activates GJs in astrocytes during brain I/R via PI3K/Akt/GSK-3β pathway.

## Methods

The animal protocol was approved by the Animal Ethics Committee of the Sun Yat-Sen University. All experiments were conducted in accordance with National Institutes of Health Guidelines for Care and Use of Laboratory Animals.

### Material

Male Sprague-Dawley (SD) rats (2 months old, 250-300 g) were provided by Experimental Animal Center of Southern Medical University, Guangdong, China. Efforts were made to minimize the number used and suffering of animals. All animals were housed under climate-controlled condition, and free to get standard rodent diet and tap water. Dexmedetomidine was from Jiangsu Heng Rui Medicine (Jiangsu, China). The yohimbine hydrochloride, 2,3,5-triphenyl-tetrazolium chloride (TTC), 4′,6-diamidino-2-phenylindole (DAPI), as well as mouse anti-Cx43 and mouse anti-β-tubulin antibodies were from Sigma (St. Louis, MO, USA). Horseradish peroxidase (HRP)-conjugated goat anti-mouse secondary antibody came from Jackson ImmunoResearch Laboratories (West Grove, Pennsylvania, USA). Rabbit polyclonal anti-Cx43 antibody, LY294002, SB216763, anti-mouse Alexa 488, and anti-rabbit Alexa 555 were from Cell Signaling Technology (Beverly, MA, USA). The mouse monoclonal anti-glial fibrillary acidic protein (GFAP) antibody was from Abcam (Cambridge, UK). Dulbecco’s phosphate-buffered saline (DPBS), fetal bovine serum (FBS) and dulbecco’s modified eagle medium (DMEM) were from Gibco (Auckland, New Zealand). 5% goat serum was from Solarbio (Beijing, China).

### Experimental protocols

In animal experiments, two-month-old rats weighed 250 g to 300 g were randomly allocated into 5 different groups according to random number table, including sham operation, right middle cerebral artery occlusion (MCAO) pretreated with normal saline, MCAO pretreated with dexmedetomidine (5 μg/kg), MCAO pretreated with yohimbine (YOH, 1 mg/kg) and MCAO pretreated with dexmedetomidine (5 μg/kg) + YOH (1 mg/kg) group (*n* = 10 per group). Animals were treated 30-min with dexmedetomidine or normal saline before MCAO through tail vein injection. Yohimbine was also administered intravenously before dexmedetomidine infusion. After 90-min occlusion, rats in MCAO groups were re-perfused for 72 h. Brains were then harvested to assess the infarct volume and expression of GFAP and Cx43.

### Transient middle cerebral arterial occlusion model

Brian ischemia was created by right MCAO as previously described [15]. Briefly, rats were intubated and mechanically ventilated with 2% sevoflurane after induction. Body temperature was maintained at 37 °C through the servo-controlled warming blanket. The heart rate and pulse oximeter oxygen saturation were monitored with MouseOX Murine Plus Oximeter System (Starr Life Sciences Corporation, Oakmont, PA, USA). After a midline cervical incision and exposure of the right common carotid artery, the external carotid artery was ligated distally. A 6–0 monofilament nylon suture (Beijing Cinontech Co. Ltd., Beijing, China) with a rounded tip was advanced from the external carotid artery into the lumen of the internal carotid artery to achieve MCAO. Sevoflurane inhalation was stopped once the suture was completed. The filament was withdrawn after 90 min of cerebral ischemia to allow reperfusion. Laser-Doppler flowmetry was used to measure cerebral blood flow during experiment. Animals with cerebral blood flow reduction > 90% or < 70% were excluded from the study. Twenty-two animals were excluded because of blood flow or death for accident before endpoint. Blood glucose levels were measured by a glucometer using the animals’ tail vein blood sample before MCAO model induction. There was no significant difference between different groups in blood glucose levels (*P* < 0.05 was regarded as statistically significant followed by One-way ANOVA). Rat’s blood pressure was monitored continuously and noninvasively. There was no obviously difference in blood pressure among different groups (*P* < 0.05 was regarded as statistically significant followed by One-way ANOVA).

### Evaluation of motor coordination, neurological deficit scores, infarct volumes and hemorrhagic volumes

The animals’ motor coordination was evaluated with the accelerating rotarod and its neurological deficit scores were measured by a special fellow blinded to the study. Neurological deficit scores were carried out according to an eight-point scale: 0, no apparent deficits; 1, failure to extend left forepaw fully; 2, decreased grip of the left forelimb; 3, spontaneous movement in all directions, contralateral circling only if pulled by the tail; 4, circling or walking to the left; 5, walking only if stimulated; 6, unresponsiveness to stimulation and with depressed level of consciousness; 7, dead [16]..

Rats were anesthetized with 8% sevoflurane for euthanasia and transcardially perfused with cold saline at 3 days after MCAO. Brains were dissected and washed in PBS, then sliced into 2 mm thick coronal sections. Infarct volumes were measured by 2% TTC staining [17, 18]. Then sections were photographed by a digital camera (M205FA, Leica, Germany) and analyzed using NIH ImageJ software (NIH, USA). The infarct volumes were calculated with the formula of total contralateral hemisphere area minus non-infarcted area of ipsilateral hemisphere. The infarct percentage was calculated as: ipsilateral infarct volumes/total contralateral hemisphere volume × 100%. The calculation of hemorrhagic volumes was performed before TTC staining and was measured the same as infarct volume.

### Immunofluorescent staining

Brains were removed, placed in 4% paraformaldehyde for 12 h, transferred to a 30% sucrose solution for 48 h, and then sliced for immunofluorescence labeling [19]. Then, sections were incubated in 0.3% Triton X-100 containing PBS (using a shaking plate, 10 times for 10 min each) and blocked with 5% goat serum for 1 h. For double immunofluorescence staining, brain slices (20 μm) were incubated with primary rabbit polyclonal anti-Cx43 antibody (1:100) and mouse monoclonal anti-GFAP antibody (1:100) at 4 °C overnight. The appropriate secondary antibodies, including anti-mouse Alexa 488, and anti-rabbit Alexa 555, were incubated with the slices for 1 h at room temperature. Nucleus were stained with DAPI for 1 h, sections were coverslipped with glycerol, and fluorescent images were obtained with a fluorescence microscope (Zeiss, Germany).

### Culture of primary astrocyte

Primary astrocytes were prepared from 1-day-old SD rats from the Animal Center of Sun Yat-Sen University. Astrocytes were isolated from the brains as described previously [20]. Briefly, brains were immediately dissociated in ice-cold DPBS. After removing the meninges and adherent blood vessels, prefrontal cortical tissue was dissected out and incubated in DMEM with 10% FBS. Tissue was triturated and centrifuged. Cell suspension was seeded and incubated in DMEM/F12 with 10% FBS, 100 U/ml penicillin and 100 mg/ml streptomycin. Culture media was replaced every 3 days. More than 95% of cells were GFAP-positive astrocytes after 12–14 days.

### OGD model of astrocyte

Oxygen-Glucose Deprivation (OGD) model was performed by the previous methods [21]. Briefly, astrocytes were washed twice and incubated in glucose-free DMEM. Then the cultures were placed in an anaerobic chamber filled with 95% N_2_/5%CO_2_ at 37 °C for 4 h. The astrocytes were returned to the normal culture condition for 48 h to reach re-oxygenation.

### Western analysis of brain tissue and astrocytes

The Fr1 area, between bregma + 2 and 0 mm, was harvested [18]. It was homogenized in buffer A (10 mM HEPES, 1.5 mM MgCl_2_, 10 mM KCl, 0.5 mM DTT, 0.05% NP40, PH 7.9) and protease inhibitors (10 mg/ml aproteinin, 5 mg/ml peptastin, 5 mg/ml leupeptin and 1 mM phenyl-methanesulfonylfluoride) as previously described [16]. After centrifuging at 12,000 rpm, 4 °C for 30 min, the supernatant was harvested. Astrocytes were scraped and lysed in a RIPA lysis buffer on ice for 1 h at 4 °C. Total and nuclear proteins were extracted and quantified using BCA kit. Lysates were solubilized in SDS sample buffer (40μg/lane) and separated by 10% SDS-PAGE (110 V, 75 min). PVDF membranes were immersed overnight at 40 °C in primary antibodies (Mouse anti-Cx43 antibody (1:10000) and mouse anti-β-Actin antibody (1:10000)) after protein separation and transfer. Secondary antibodies (1:10000) were used to detect the immunoreactivity. Quantitative analysis of the bands was performed with the Image-Quant 5.0 GE Healthcare Densitometer (GE Healthcare, Sunnyvale, CA, USA). The density of Cx43 bands were normalized with β-tubulin proteins for the same sample.

### Enzyme-linked immuno sorbent assay (ELISA)

Brain tissue in Fr1 area was homogenized and centrifuged at 12,000 rpm for 20 min. The supernatant was collected and stored at − 20 °C until use. IL-1β, IL-6 and TNF-αELISA kits (Boster Biotechnology Co., Ltd., Wuhan, China) were used for detection.

### Flow cytometry of cell

To determine the apoptosis of astrocytes, flow cytometry analyses were performed by Annexin V-FITC apoptosis detection kits (Biotool, Houston, TX, USA) [22]. After dispose of OGD for 4 h and re-oxygenation for 48 h, cellular apoptosis was measured by FACS (BriCyte E6, Mindray, China). Data was analyzed through the FlowJo software (Treestar, Inc., San Carlos, California, USA) and illustrated via percentages and dot images.

### Statistical analysis

SPSS statistical software (SPSS 24.0) was used to perform statistical analyses and the parametrical data were presented as mean ± SD. Data among multiple groups including speed–latency index ratio, infarct volume, western blotting, ELISA and Cx43 immunofluorescent staining was analyzed by One-way ANOVA, followed by Tukey’s test. Neurologic deficit scores were analyzed by one-way ANOVA on ranks, followed by the Tukey’s test. *P* < 0.05 was regarded as statistically significant.

## Results

### Dexmedetomidine reduces lesion size and enhances neurological function after brain I/R

**Fig. 1:**
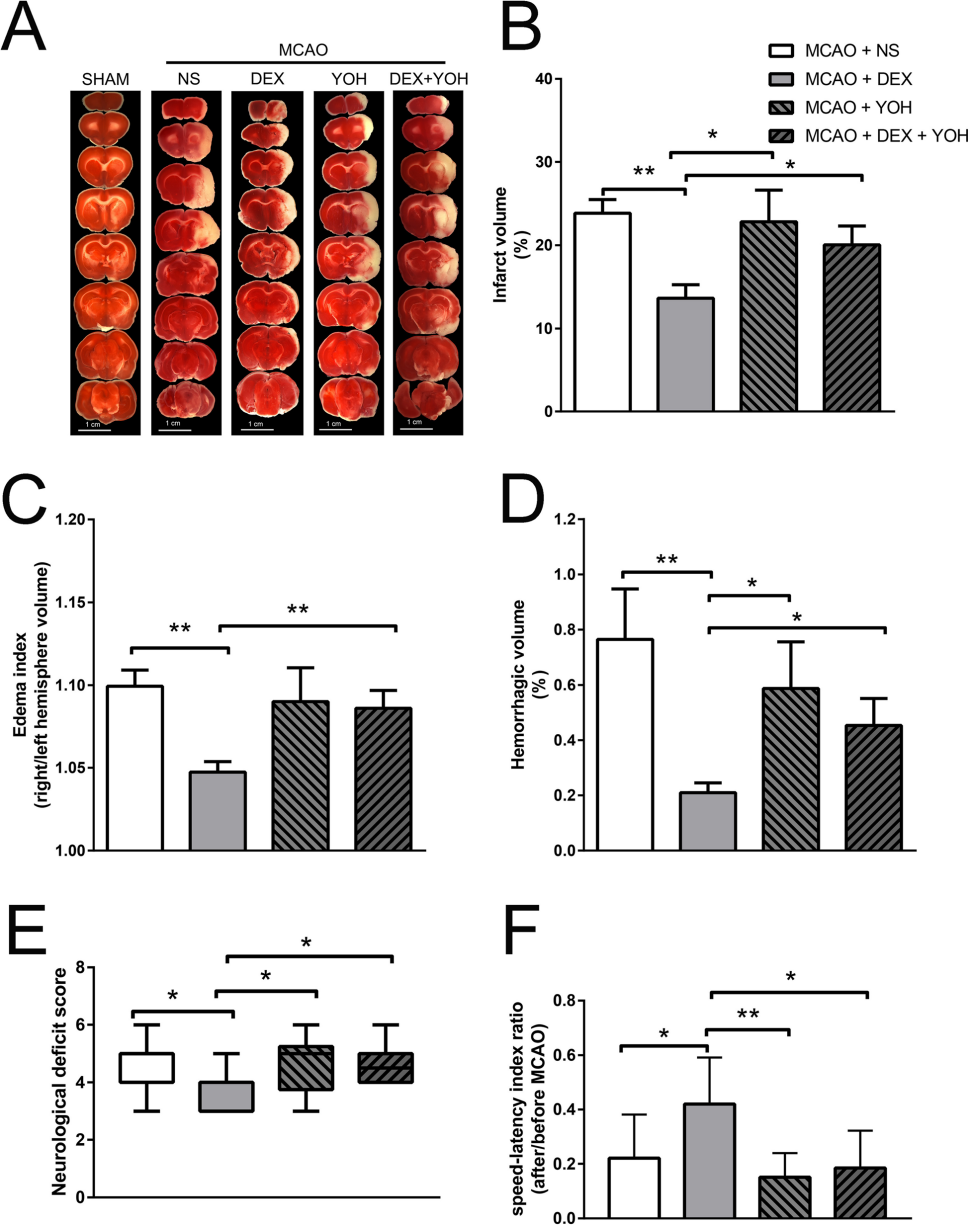
Dexmedetomidine-induced neuroprotection after brain I/R. **a** Rats were pretreated with normal saline, dexmedetomidine or yohimbine before transient middle cerebral artery occlusion (MCAO, 90 min occlusion). Brain sections were achieved and staining with TTC after 72 h reperfusion. **b** Percentage of infarct volume of right hemisphere over ipsilateral hemisphere. **c** Edema index was calculated though right/left hemisphere volume. **d** Percentage of hemorrhagic volume of right hemisphere over ipsilateral hemisphere. **e** Neurological deficit scores. Between lines, 95% interval of the data; inside boxes, 25–75% interval. **f** Rats were tested before and 3 days after the MCAO and the speed–latency index ratio of these two tests were analyzed. Results except panel E were presented with mean ± SD (*n* = 10) and analyzed using One-way ANOVA followed by Tukey’s multiple comparisons test. *P < 0.05, **P < 0.001. NS, normal saline. DEX, dexmedetomidine. YOH, yohimbine. TTC, 2,3,5-triphenyl-tetrazolium chloride

**Fig. 2:**
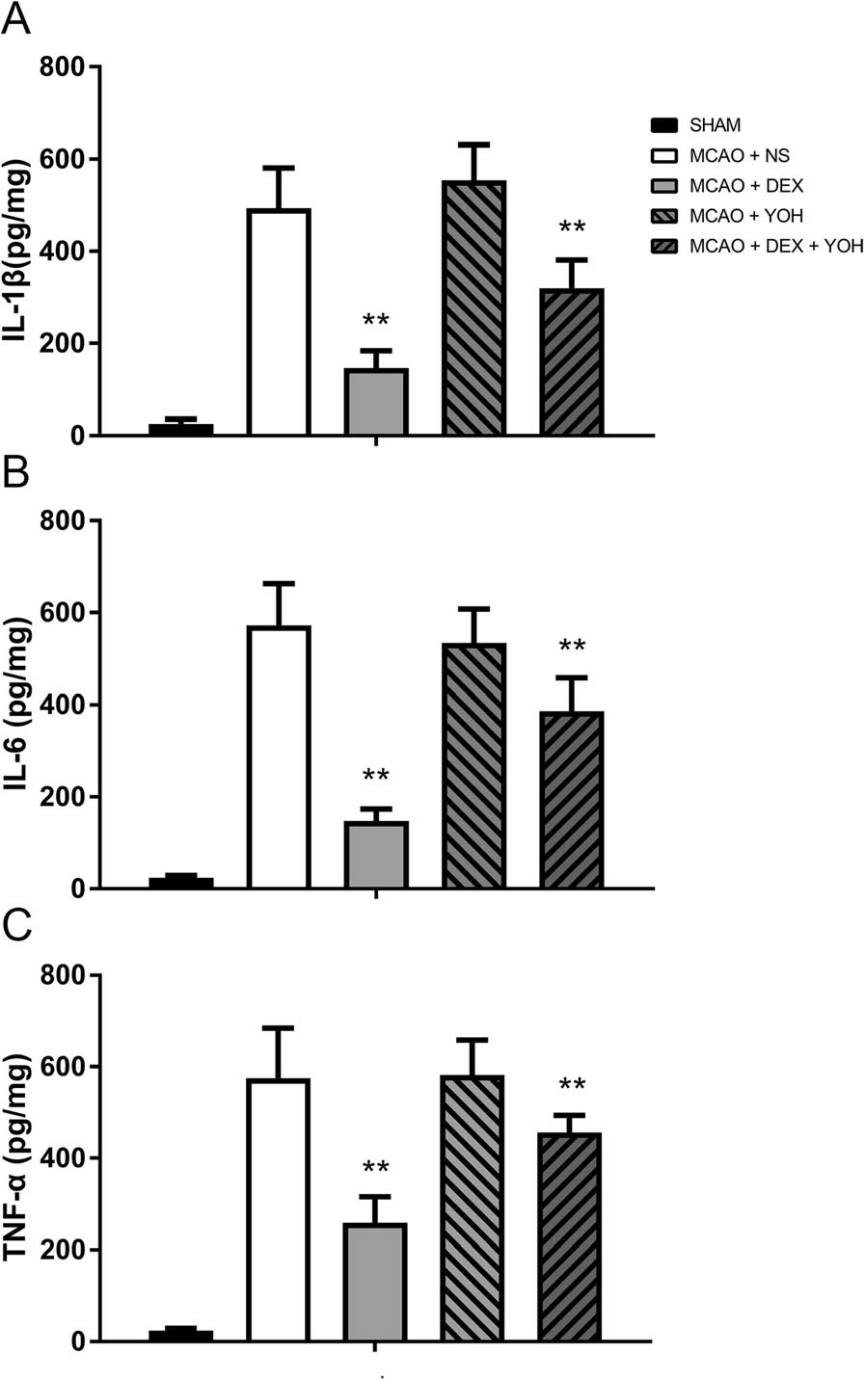
Dexmedetomidine attenuated release of pro-inflammatory factors after brain I/R. After reperfusion for 72 h, ischemic brain tissue was harvested to assess the release of IL-1β (**a**), IL-6 (**b**), TNF-α (**c**) through ELISA kits. Results of B-D were presented with mean ± SD (*n* = 10) and analyzed using One-way ANOVA followed by Tukey’s multiple comparisons test. **P < 0.001 between MCAO+DEX and MCAO+NS or between MCAO+DEX + YOH and MCAO+DEX. NS, normal saline. DEX, dexmedetomidine. YOH, yohimbine. TTC, 2,3,5-triphenyl-tetrazolium chloride

Dexmedetomidine pretreatment significantly attenuated brain lesions (infarct volume, brain edema and hemorrhage volume) caused by right middle cerebral artery occlusion (MCAO) in 2,3,5-triphenyl-tetrazolium chloride (TTC) staining, compared with MCAO+ normal saline (NS) group (*P* < 0.001) (Fig. 1a-d). Meanwhile, it also improved rotarod performance and neurological deficit scores (*P* < 0.05) (Fig. 1e, f). Measurement of the ischemic penumbral tissues showed that pro-inflammatory factors (IL-1β, IL-6 and TNF-α) increased after MCAO, which were attenuated by dexmedetomidine preconditioning (*P* < 0.001) (Fig. 2). And yohimbine, the α2-adrenergic receptors antagonist, did not affect MCAO-induced brain lesions, neurological function, and release of pro-inflammatory factors. However, yohimbine combined with dexmedetomidine down-regulated the effects of dexmedetomidine on MCAO (*P* < 0.05) (Fig.1 and 2).

### Dexmedetomidine attenuates the reduction of CX43 and the increase in apoptosis caused by OGD and re-oxygenation of cultured astrocytes

**Fig. 3:**
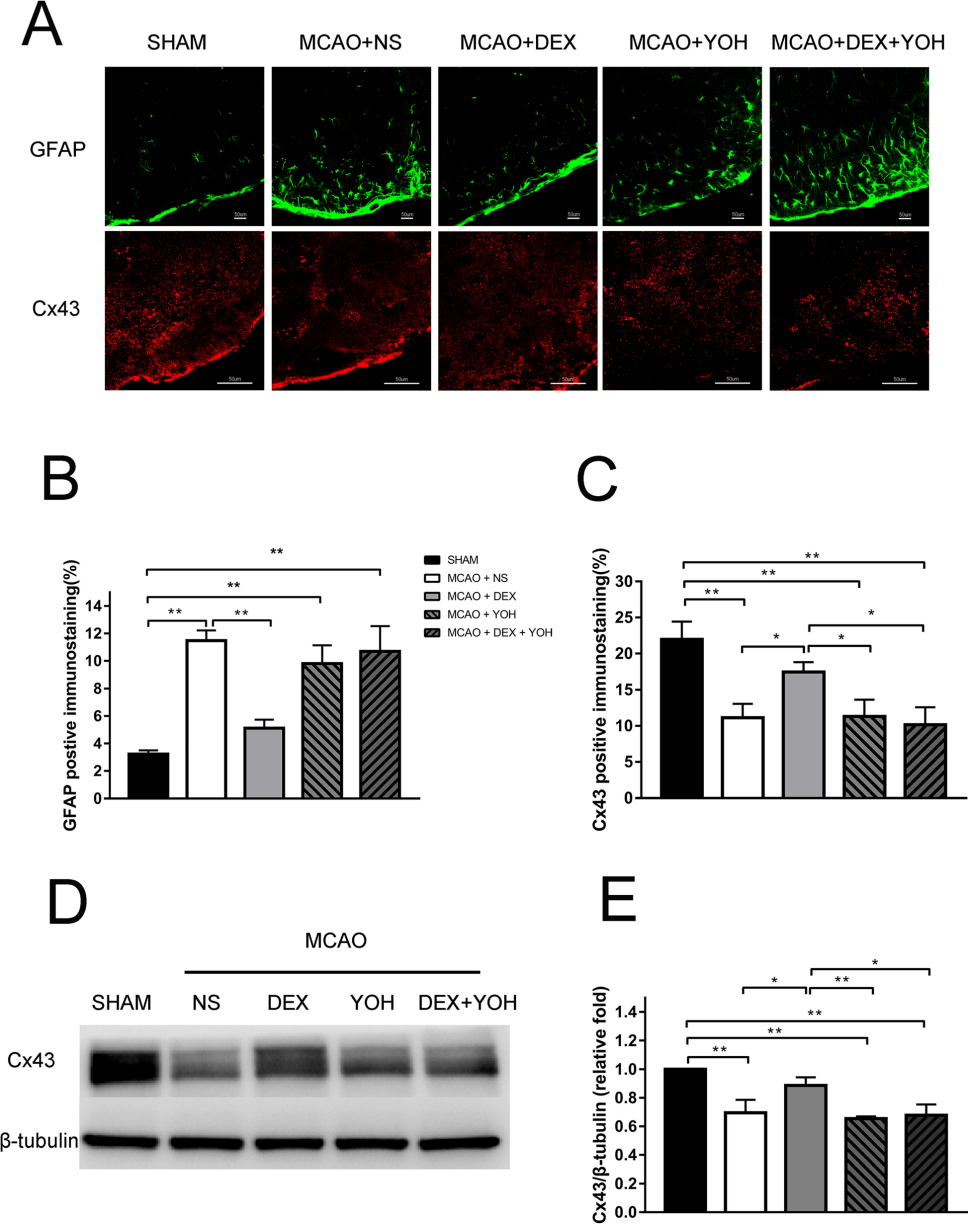
Dexmedetomidine altered brain I/R-induced increase of GFAP expression and Cx43 reduction. Brain sections of Fr1 area were harvested 72 h after 90 min middle cerebral artery occlusion for immunostaining of GFAP and Cx43. **a** Representative of immunostaining images. **b**-**c** Graphic presentation of the percentage area that is GFAP and Cx43 positive in the ischemia-adjacent area. **d** Representative Western blot image for Cx43 expression in Fr1 area. Tubulin was used as the internal loading control. **e** Graphic presentation of Cx43 protein abundance. Results were presented as mean ± SD (*n* = 5) and analyzed using One-way ANOVA followed by Tukey’s multiple comparisons test. *P < 0.05, **P < 0.001. NS, normal saline. DEX, dexmedetomidine. YOH, yohimbine. GFAP, glial fibrillary acidic protein. Cx43, Connexin 43. MCAO, middle cerebral artery occlusion

**Fig. 4:**
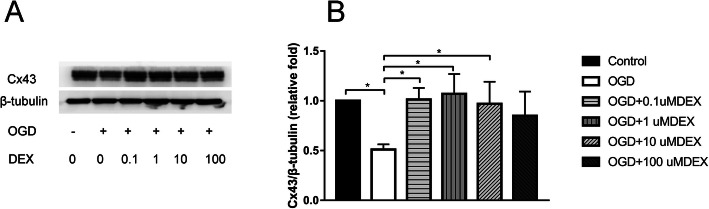
Effects of dexmedetomidine on Cx43 expression after oxygen-glucose deprivation (OGD 4 h, re-oxygenation 48 h). **a** The expression of Cx43 was assessed using Western blot and the representative image was shown. Tubulin was used as the internal loading control. **b** Quantitative analysis of each group was conducted. Results were presented as mean ± SD (*n* = 6) and analyzed using One-way ANOVA followed by Tukey’s multiple comparisons test. *P < 0.05

**Fig. 5:**
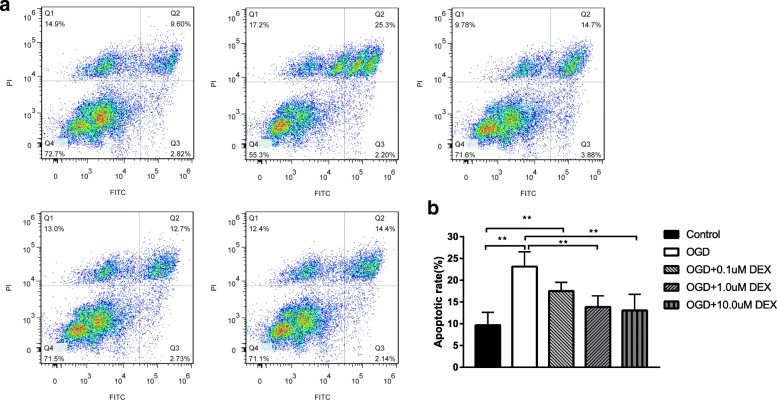
Effects of dexmedetomidine on early-stage apoptosis induced by oxygen-glucose deprivation (OGD 4 h, re-oxygenation 48 h). **a** Early-stage apoptosis was assessed by flow-cytometry. **b** Relative quantity of early-stage apoptosis ratio was present. Results were presented as mean ± SD (*n* = 6) and analyzed using One-way ANOVA followed by Tukey’s multiple comparisons test. **P < 0.001. OGD, oxygen-glucose deprivation

Fr1 area of brain tissue was extracted to measure the response of astrocyte GJs to brain I/R. The elevated glial fibrillary acidic protein (GFAP) (Fig. 3a, b) and decreased Cx43 (Fig. 3a, c) were observed in the ischemic penumbra area of MCAO by immunofluorescent staining, compared with sham (*P* < 0.001). Western blot analysis confirmed the marked inhibition of Cx43 expression in Fr1 area of MCAO than sham rats (*P* < 0.001) (Fig. 3d, e). Cx43 was reduced in oxygen-glucose deprivation (OGD) group, concordant with in vivo results (*P* < 0.05) (Fig. 4a-b). In vitro, dexmedetomidine (from 0.1-100 μM) was adopted on astrocyte for 1 h before OGD and induced the up-regulation of Cx43 as shown by Western blot (*P* < 0.05) (Fig. 4a-b). However, a decreasing tendency of Cx43 increase was observed with dexmedetomidine reaching the concentration of 10-100 μM (Fig. 4a-b). Flow-cytometry showed that dexmedetomidine (from 0.1-10 μM) could attenuate OGD-induced astrocytes apoptosis (*P* < 0.001) (Fig. 5).

### Dexmedetomidine altered astrocytes GJs expression through PI3K/Akt/GSK-3β pathway in OGD model

**Fig. 6:**
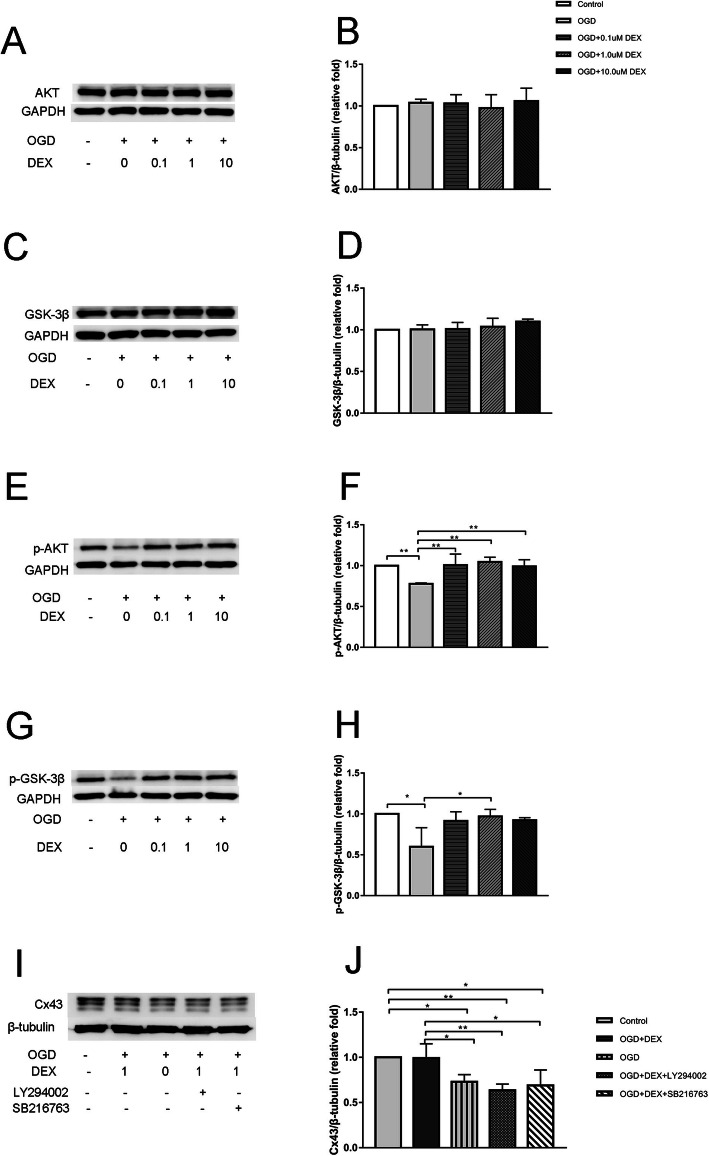
Dexmedetomidine modulated OGD-induced Cx43 reduction through PI3K-Akt-GSK-3β pathway. **a**, **c**, **e**, **g** Western blot of PI3K-Akt-GSK-3β signaling pathway. Representative images were shown. **b**, **f** Quantitative analysis of Akt and p-Akt expression. **d**, **h** Quantitative analysis of GSK-3β and p-GSK-3β expression. **i** Primary astrocytes were incubated with LY294002 or SB216763 for 1 h before OGD, then Cx43 was examined using Western blot. **j** Quantitative analysis of Cx43 expression with LY294002 or SB216763 pretreatment. Results were presented as mean ± SD (n = 6) and analyzed using One-way ANOVA followed by Tukey’s multiple comparisons test. *P < 0.05, **P < 0.001. LY294002, PI3K inhibitor. SB216763, GSK-3β inhibitor

The alteration of PI3K/Akt/GSK-3β pathway was tested in OGD model. 4 h deoxygenation and 48 h re-oxygenation or dexmedetomidine pretreatment didn’t influence expression of total Akt or GSK-3β (Fig. 6a-d). However, after 4 h deoxygenation and 48 h re-oxygenation, the decreased expression of p-Akt and p-GSK-3β than control was observed (*P* < 0.05) (Fig. 6e-h). Pretreatment with dexmedetomidine in the concentrations (from 0.1-10 μg/ml) for 1 h inhibited the above changes of p-Akt and p-GSK-3β in a dose-independent manner (*P* < 0.05) (Fig. 6e-h). But in p-GSK-3β, dexmedetomidine only in the concentration of 1μg/ml could produce statistically significant result (Fig. 6g-h).

### Inhibiting PI3K or GSK-3β eliminated dexmedetomidine effects on GJs alteration in vitro

Pre-incubation with LY294002 (PI3K inhibitor) or SB216763 (GSK-3β inhibitor) significantly blocked the up-regulation of Cx43 induced by dexmedetomidine (*P* < 0.05) (Fig. 6i-j).

## Discussion

In the current study, we demonstrated that dexmedetomidine attenuated the neurotoxicity against brain I/R injury in vivo and vitro. This process correlated with the up-regulation of astrocyte Cx43 through phosphorylation of PI3K/Akt/GSK-3β pathway in OGD model.

### Dexmedetomidine may alleviate brain ischemia and reperfusion injury by upregulating Cx43

Ischemia is one of the causes of cells death induced by regional cerebral hypoperfusion, following with the accumulation of spatial toxic molecules and inflammatory cascade. Ischemia-reperfusion (I/R) injury occurs when blood resupply after a period of ischemia time. During this process, brain GJs may be effector molecules as they mediate electrochemical communication between cells. Brain GJs are formed by the interactions of two hemichannels on adjacent cells, connecting the cytoplasm between them. The hemichannel is developed by six connexin monomers, which is characterized as membrane protein, and functions as transmitter release pores [23]. Cx43 is the most abundant connexin in the brain, which is prominently expressed in astrocytes [24]. Under normal condition, astrocyte Cx43 establishes a communication between neurons and astrocytes, for the elimination of toxic molecules in neurons. During brain ischemia, Cx43 gets internalized and decreased, which is believed to block the spreading of injury to surrounding healthy cells [25]. The regulation of astrocyte Cx43 might plays a pivotal role against brain I/R injury pathogenesis, as indicating by previous studies [26–28]. However, there are still no effective methods to modulate their alteration status.

In our study, in vivo and in vitro environment were applied to imitate brain I/R injury. MCAO rat model was successfully established with significantly increased infarct volume in the ipsilateral perfusion area of MCAO. OGD model of astrocyte was confirmed success through the markedly increased apoptosis of cells. Cx43 was decreased in response to ischemia/reperfusion in our research while some studies found Cx43 increasing in post-ischemia [29]. Meanwhile, IL-1β, IL-6 and TNF-α were markedly increased in brains after ischemia/reperfusion. These are consistent with the previous studies [16]. But more research should be carried out to find out that whether dexmedetomidine alleviated inflammation reaction directly or through upregulating Cx43.

Dexmedetomidine is an α2 adrenoceptor agonist with sedative and analgesic effects. Studies suggested that it could improve neurological and histopathological outcomes against brain ischemia. The inhibition of inflammation and sympathetic activity might be its potential mechanisms [30]. In this study, we found that dexmedetomidine alleviated brain lesions and neurological dysfunction against brain I/R injury. Also, a decreased apoptosis of astrocytes in vitro was found. Meanwhile, proinflammatory factors were significantly decreased by dexmedetomidine. However, it is interesting that this protective effect was partially inhibited by yohimbine, an α2-adrenergic receptors antagonist. We suggested that the effects of dexmedetomidine may be mediated at least partially through receptors or molecules other than the a2 adrenoceptor.

Then, we assessed the effects of dexmedetomidine on astrocyte Cx43 after I/R. The specific effect of dexmedetomidine on connexins remains controversial. Previous studies suggested that dexmedetomidine had little inhibitory effect on lipopolysaccharide (LPS)-induced astrocyte connexin channel function [7]. Other research showed that it could reduce LPS-induced apoptosis through suppressing the function of Cx43-composed GJs in lung fibroblasts [31]. In our study, we found that dexmedetomidine administration induced astrocyte Cx43 expression, both in vivo and in vitro. In addition, vitro study demonstrated that low dose of dexmedetomidine (0.1 μM) could exhibit a significantly effect on the upregulation of astrocyte Cx43. However, this effect showed a decreasing trend with a higher concentration (100 μM), without reaching statistical significance. These results were partially consistent with previous studies that higher concentration of dexmedetomidine exhibited an inhibitory action [7]. We suggest that dexmedetomidine-induced negative effects at an excessive dosage may have nonspecific effects. In addition, our research is the first to demonstrate the up-regulatory effect of dexmedetomidine on astrocyte Cx43 in lower dosage.

### PI3K/Akt pathway involved in the mechanism of dexmedetomidine in regulating Cx43 in OGD

The co-occurrences of reducing brain I/R injury and astrocyte Cx43 up-regulation after dexmedetomidine provide grounds for our hypothesis that dexmedetomidine might attenuate brain I/R injury through astrocyte Cx43 up-regulation. To further illustrate the specific mechanisms, we evaluated the potential pathways on astrocyte Cx43. PI3K/Akt pathway is believed to be neuroprotective during brain ischemia. Akt is a serine/threonine kinase and it acts by mTOR and PDK1 [32]. Activation of Akt is involved in cell survival during ischemia. GSK-3β, the downstream of Akt, is highly expressed in brain. It is phosphorylated by Akt at Ser9, resulting its inactivation [33]. Phosphorylation of GSK-3β has been proved to inhibit propofol-induced lysosome/mitochondrial apoptosis in macrophages [14, 34]. Meanwhile, the increase of Cx43 expression in cardiomyocytes has been revealed to be regulated by Akt and GSK-3β [35]. In the current study, we observed the inhibition of p-Akt at Ser473 and p-GSK-3β at Ser9 after OGD. Pretreatment with dexmedetomidine significantly reversed their inhibitory changes. These suggested the possible mediating role of PI3K/Akt/GSK-3β pathways on the protective effects of dexmedetomidine. In order to evaluate this function, we further modulated these pathways with their inhibitors (PI3K inhibitor: LY294002 or GSK-3β inhibitor: SB216763) and results showed their intrinsic effects on Cx43 expression. These results suggest that dexmedetomidine can up-regulate astrocyte Cx43 through the activation of PI3K/Akt/GSK-3β pathways in vitro.

In our study, dexmedetomidine administration up-regulated astrocyte Cx43 might be the potential direction of future therapy for brain I/R injury. Our findings have some conflicts with previous understanding that down-regulation of GJs is essential for alleviation of brain I/R injury [24]. While consistent to our results, previous studies applying heterozygous Cx43 knockout mice showed a significantly increasing infarct volume and decreasing astrogliosis after MCAO [27, 36].

### Limitation

In our study, higher expression of Cx43 was not equal to the activation of GJs, thus more studies should be applied to figure out the different influence between Cx43 and GJs when exposing to dexmedetomidine. The relationship between up-regulating astrocyte Cx43 and GJs function, as well as neurons survival after brain I/R injury requires further exploration. Meanwhile, as it was recently demonstrated that reactive astrocyte had two phenotypes, A1 and A2. They mediated neurotoxic and neurotrophic effect respectively [37]. The up-regulation of Cx43 by dexmedetomidine might function as an intermediate molecule to induce the transformation of A2 phenotype, and subsequently attenuate brain I/R injury.

## Conclusion

In conclusion, dexmedetomidine plays an important role in the modulation of astrocyte Cx43 after brain I/R injury through the upregulation of PI3K/Akt/GSK-3β. It tends to reduce brain injury after I/R via the upregulation of astrocyte Cx43. Therefore, astrocyte Cx43 might be the potential therapeutic target for brain I/R injury during perioperative period. Further studies are needed to understand the role of Cx43 in I/R injury.

## Supplementary Information

**Additional file 1.**

## Data Availability

The datasets used and/or analysed during the current study are available from the corresponding author on reasonable request.

## Abbreviations

Cx43: Connexin 43
I/R: Ischemia reperfusion
MCAO: Middle cerebral arterial occlusion
OGD: Oxygen-glucose deprivation
GJs: Gap junctions
ROS: Reactive oxygen species
PI3K: Phosphatidylinositol-4,5-bisphosphate 3-kinase
GSK-3β: Activating glycogen synthase kinase-3β
TTC: 2,3,5-triphenyl-tetrazolium chloride
DAPI: 4′,6-diamidino-2-phenylindole
GFAP: Glial fibrillary acidic protein
DPBS: Dulbecco’s phosphate-buffered saline
FBS: Fetal bovine serum
DMEM: Dulbecco’s modified eagle medium
YOH: Yohimbine
